# EPIC: Annotated epileptic EEG independent components for artifact reduction

**DOI:** 10.1038/s41597-022-01524-x

**Published:** 2022-08-20

**Authors:** Fábio Lopes, Adriana Leal, Júlio Medeiros, Mauro F. Pinto, António Dourado, Matthias Dümpelmann, César Teixeira

**Affiliations:** 1 University of Coimbra, Center for Informatics and Systems of the University of Coimbra, Department of Informatics Engineering, 3030-290 Coimbra, Portugal; 2 Epilepsy Center, Medical Center – University of Freiburg, Department Neurosurgery, Faculty of Medicine, University of Freiburg, 79106 Freiburg, Germany

**Keywords:** Databases, Biomedical engineering

## Abstract

Scalp electroencephalogram is a non-invasive multi-channel biosignal that records the brain’s electrical activity. It is highly susceptible to noise that might overshadow important data. Independent component analysis is one of the most used artifact removal methods. Independent component analysis separates data into different components, although it can not automatically reject the noisy ones. Therefore, experts are needed to decide which components must be removed before reconstructing the data. To automate this method, researchers have developed classifiers to identify noisy components. However, to build these classifiers, they need annotated data. Manually classifying independent components is a time-consuming task. Furthermore, few labelled data are publicly available. This paper presents a source of annotated electroencephalogram independent components acquired from patients with epilepsy (EPIC Dataset). This dataset contains 77,426 independent components obtained from approximately 613 hours of electroencephalogram, visually inspected by two experts, which was already successfully utilised to develop independent component classifiers.

## Abstract (summary)

**Table:** 

Measurement(s)	Electroencephalogram Independent Components
Technology Type(s)	MATLAB and Python

## Background & Summary

Scalp electroencephalogram (EEG) records electrical activity generated in the brain. It is a multi-channel biosignal obtained using non-invasive acquisition systems^1,2^. Being a non-invasive biosignal, EEG is usually contaminated with artifacts such as eye blinks, eye saccades, and muscle activity^3–5^. These artifacts are characterized by a spectrum which may overlap frequencies of interest. Therefore, researchers have used different algorithms to attenuate the impact of these artifacts^6–14^. One of the most used algorithms is independent component analysis (ICA)^4,15^.

ICA is a linear blind source separation (BSS) technique that decomposes multi-channel signals into independent components (ICs)^15^. Researchers usually use ICA to obtain ICs and to generate cleaner EEG signals. However, ICA is unable to automatically reject noisy ICs. Therefore, they must be inspected by experts aiming to remove the ones related to artifacts and thus, reconstructing the EEG data with the remaining, assumed to contain brain information. Although this process usually performs well in removing noise from EEG signals, it requires visual inspection of data, making it difficult when experts are not available. Consequently, several researchers have developed classifiers to automatically perform this task^12,16–27^.

Developing classifiers to automatically label ICs requires annotated datasets. To the best of our knowledge, only Winkler *et al*.^17^ and Pion-Tonachini *et al*.^12^ made their datasets publicly available. Winkler *et al*. provided a training set with 690 ICs collected from a set of 23 recordings with 10 minutes of EEG and a test set with 1080 ICs collected from 36 EEG recordings. Data were collected from 12 subjects who had to perform provided tasks, during approximately 5 hours, avoiding producing artifacts. Both sets were classified by experts. However, they only released sets with the best six features: current density norm, range within pattern, mean local skewness of 15-second intervals, and two parameters obtained from comparing the IC spectrum with a prototypical 1/frequency curve. Pion-Tonachini *et al*. provided dataset containing ICs collected from 5-second EEG recordings acquired from several studies over the past 15 years. These studies were performed in controlled environments where the subjects performed provided tasks. The dataset is divided in training and test sets. Training set comprises 5,937 ICs classified by several collaborators through a crown labelling task. Test set includes 130 ICs labelled by seven experts. ICs were annotated with seven different classes: brain, eye, muscle, heart, channel noise, line noise, and other. Each sample is composed of scalp topographic map, power spectrum density (PSD), autocorrelation function, equivalent current dipole fits, and hand-crafted features. Although this dataset provided scalp topographic maps and PSDs, it fails at providing IC time-series. Therefore, new researchers that would like to use the data are restricted to temporal features contained in the dataset which might limit their approaches.

To the best of our knowledge, currently available datasets only contain data collected in controlled environments and do not provide all the information about ICs. As a consequence of this data restriction, we created a dataset with ICs from EEG data collected from patients with epilepsy (EPIC dataset). It comprises ICs extracted from 19-channel EEG signals available in EPILEPSIAE database^28^. Despite only providing data from patients with epilepsy, EPILEPSIAE database comprises data collected over several days from patients under pre-surgical monitoring. Therefore, data contain several day-to-day artifacts such as conversation, eating, and sleeping. Each sample includes the time-series, power spectrum density, and topographic map of each IC. Furthermore, these samples were classified as brain or noise by two experts. EPIC dataset contains a training set with 61,092 samples and a test set with 16,334 samples. Training set contains 43,038 (70.44%) brain ICs and 18,054 (29.56%) artifact ICs whereas testing set contains 11,437 (70.02%) brain ICs and 4,897 (29.98%) artifact ICs. These data were already used in a previous study^27^. In that study, authors concluded that using the three sources of information improved the IC classifiers’ performance. Furthermore, the study also showed that previously trained IC classifiers could be used to improve the performance of new IC classifiers using transfer learning.

We provide EPIC dataset to allow other researchers to develop new IC classifiers or to benchmark existent IC classification approaches. The dataset is available at 10.5281/zenodo.6620655.

## Methods

Long-term electroencephalogram (EEG) data were retrieved from the EPILEPSIAE database. These data were obtained from 25 patients with epilepsy (13 males and 12 females, aged 39.6 ± 16.8 years) during presurgical monitoring over several days at Universitätsklinikum Freiburg. Data were acquired using a sampling rate of 256 Hz and 19 electrodes organised according to the 10–20 international system^29^. Information about the access of the EPILEPSIAE database can be found on: http://epilepsy-database.eu/. Details about the process of licensing and financial contribution to maintain the database can be requested via the e-mail address provided on the web site.

Data were curated in the context of epileptic seizure prediction. To develop our seizure prediction models, we considered data ranging from 4.5 hours before the beginning of the leading seizure^30^ until its onset. This selection was performed considering that EEG data within this interval contain both normal and pre-seizure brain states^31,32^. Data were collected over several days (accounting for 684 hours of EEG signal). Typical activities were captured in the signals such as conversation, eating, drinking, washing, and sleeping. Therefore, it may contain several experimental errors such as poor electrode connection and adhesion issues, which must be minimised before performing independent component analysis (ICA). In the next section, we present the algorithm used to remove experimental errors. Subsequently, in section Independent Components Classification, we describe how the manual labelling of the independent components (ICs) was performed. It should be noted that both steps were also conducted, in a previous study, to prepare EEG data in order to train deep convolutional neural networks to automatically remove artifacts from EEG data^14^.

### Removal of experimental errors

**Fig. 1:**
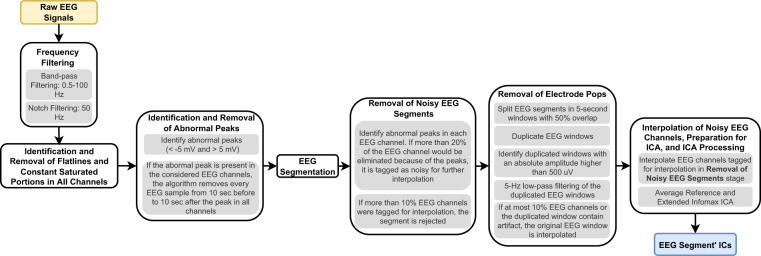
Framework followed to obtain the independent components for each EEG segment. It covers the following steps: extraction of signals from EPILEPSIAE database; identification and removal of flatlines, constant saturated portions, and abnormal peaks; EEG segmentation; removal of noisy EEG segments; removal of electrode pops; and interpolation of noisy EEG channels and preparation for ICA.

We developed an algorithm to identify and remove data corrupted by experimental errors. Figure 1 provides a diagram explaining it. The methods used in the algorithm are ordered by the simplest to the most complex one. Therefore, it consists in frequency filtering, identification and removal of flatlines and constant saturated portions in all channels, identification and removal of abnormal peaks, EEG segmentation, removal of noisy EEG segments, removal of electrode pops, and interpolation of noisy EEG channels, preparation for ICA, and ICA processing.

#### Frequency filtering

The algorithm filtered the data using a 0.5–100 Hz bandpass 4th-order Butterworth filter and a 50 Hz 2nd-order notch filter with the purpose of removing the DC component, high-frequency noise, and the powerline interference.

#### Identification and removal of flatlines and constant saturated portions in all channels

**Fig. 2:**
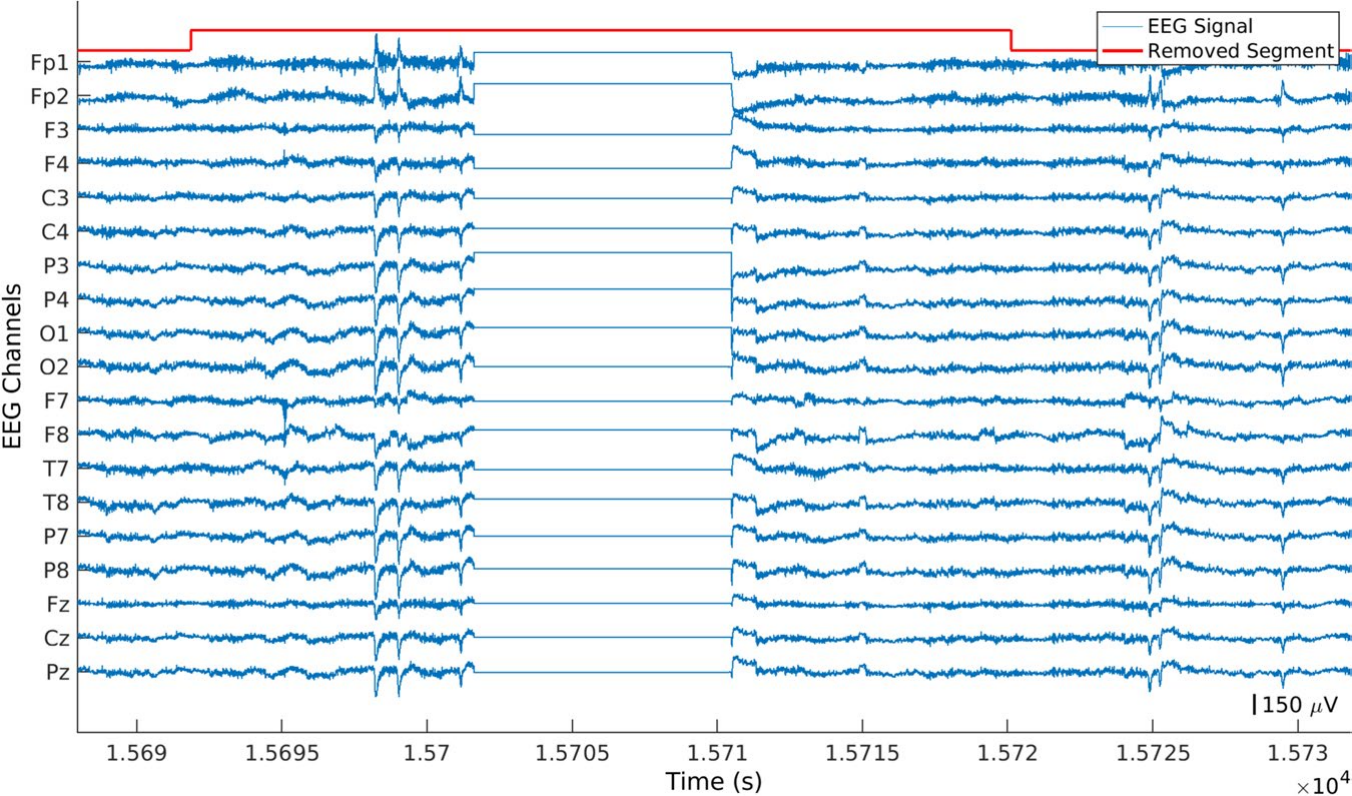
Example of an EEG signal with a flat segment. The selected portion was removed over all channels.

**Fig. 3:**
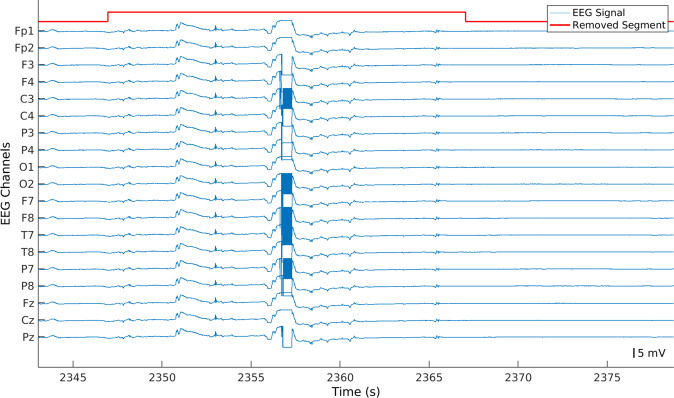
Example of an EEG signal containing a saturated segment. The selected portion was removed over all channels.

Since the data were collected over several days including day-to-day activities, these contained several experimental errors. Our algorithm identified and removed every portion of the signal which contained isoelectric flatlines (see Fig. 2) or constant saturated segments (see Fig. 3) as well as the 10 seconds of data before and after these errors. These errors were removed for all channels simultaneously.

#### Identification and removal of abnormal peaks

**Fig. 4:**
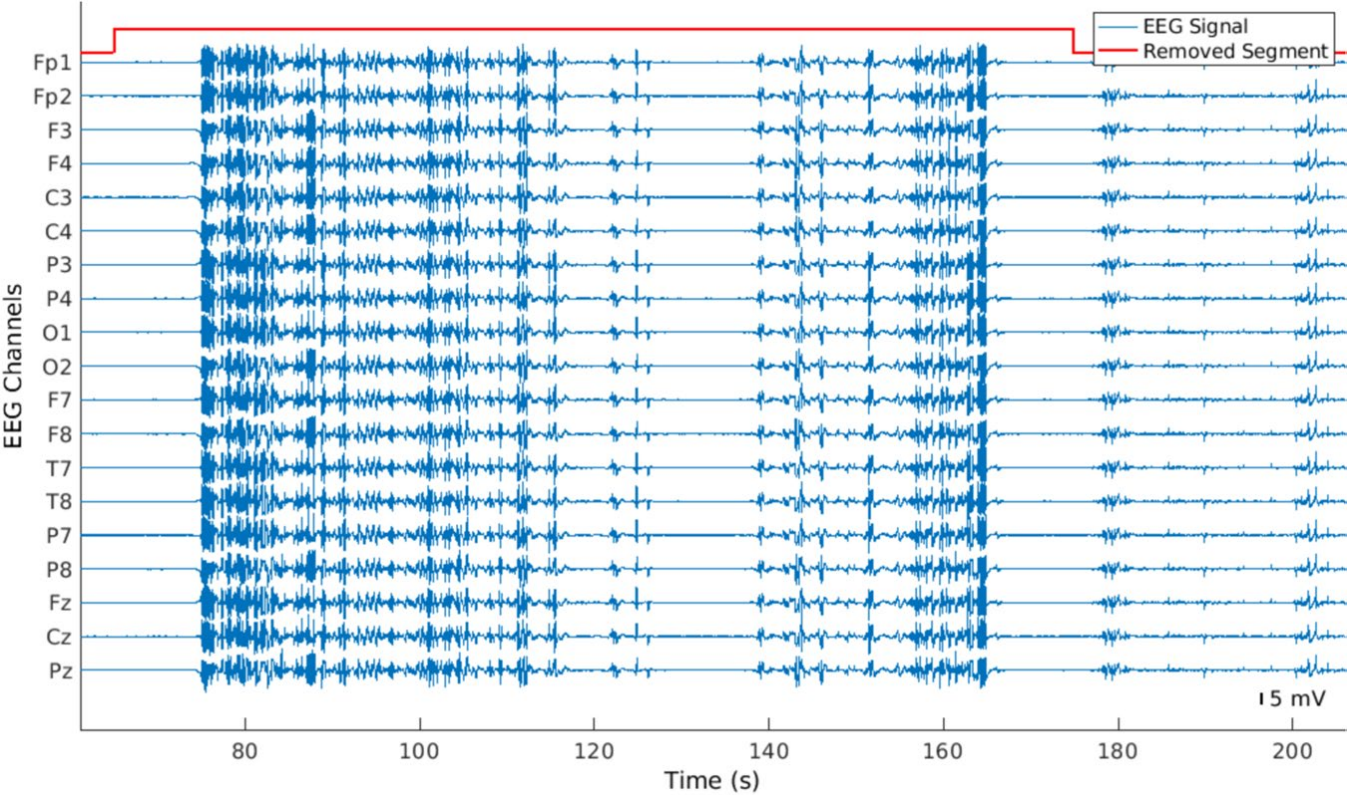
Example of an EEG signal with several abnormal peaks. As these artifacts appeared in Fp1, Fp2, T7, T8, O1, O2, and Cz, the selected EEG portion was removed across all channels.

**Fig. 5:**
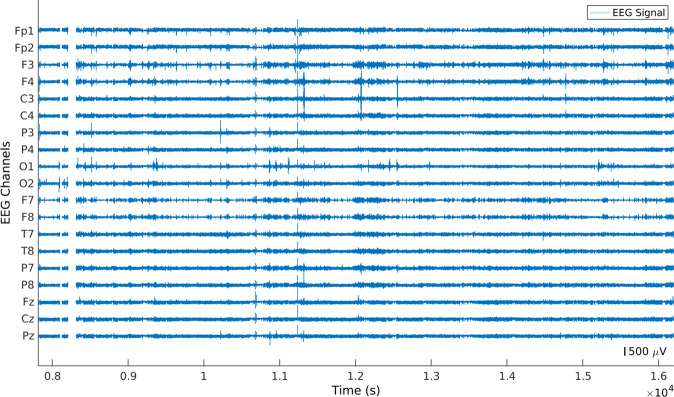
Example of a preprocessed EEG signal. The EEG segments were not concatenated after removing noisy data.

After the removal of flat and constant segments, the algorithm identified portions of the signal below −5 mV and above 5 mV, which we named abnormal peaks for easier comprehension (see Fig. 4). Then, the algorithm verified whether the peaks happened in the channels Fp1, Fp2, O1, O2, T5, T6, and Cz, at the same time. These electrodes were selected according to their geometrical positions, which means that if an abrupt movement affected the system, all of them should capture it. If abnormal peaks appeared in all the aforementioned electrodes, every sample, from 10 seconds before the beginning of the peak until 10 seconds after the peak, was removed. It is worth noting that, in order to keep the temporal coherence of the signals, we did not concatenated the data after removing artifacts (see Fig. 5).

#### EEG segmentation

**Fig. 6:**
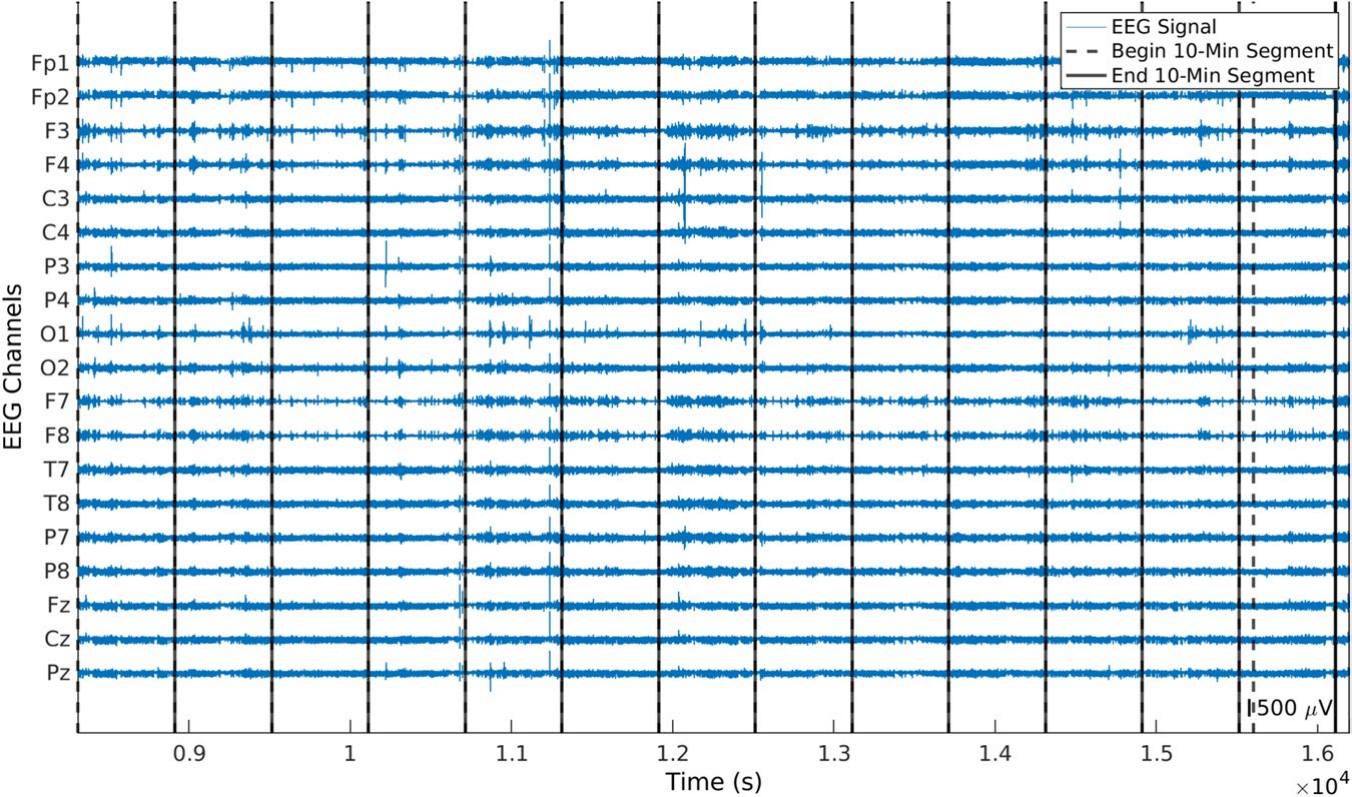
Example of a preprocessed EEG signal divided into 10-minute segments. Dashed and continuous lines determine the beginning and the end of the 10-minute segments, respectively. The last segment contains samples from the previous one because the duration of the subsignal is not divisible by 10.

The algorithm divided the remaining data in 10-minute segments (see Fig. 6). The segmentation in 10-minute portions was performed to prepare the data for the ICA^22,24^. As the signals were not concatenated after removing errors, there might be segments lasting less than 10 minutes. Despite lasting less than 10 minutes, these segments were kept to obtain the largest possible dataset. The algorithm removed every segment lasting less than 10 seconds as these did not comprise enough data to be properly processed by ICA.

#### Removal of noisy eeg segments

After the EEG segmentation, the algorithm still could identify abnormal peaks which remained in the EEG channels. If more than 20% of the EEG channel would be removed as a consequence of having abnormal peaks, then the channel was marked to be interpolated. The segment is rejected if more than two EEG channels were marked for interpolation (>10% of all EEG channels).

#### Removal of electrode pops

**Fig. 7:**
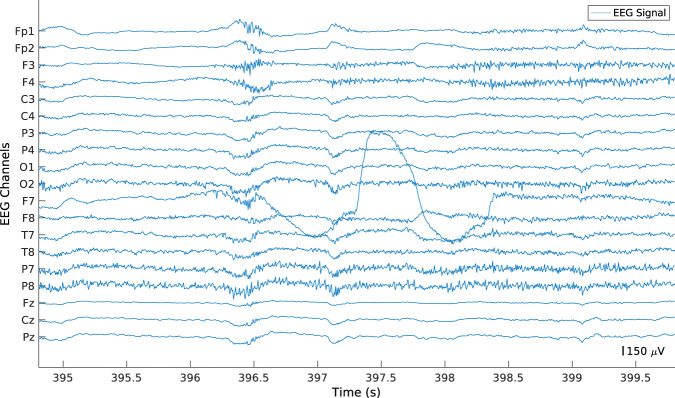
Example of EEG segment with an electrode pop present in channel F7.

At this point, the algorithm divided the segments into 5-second windows with a 50% overlap. After that, the algorithm filtered the windows using a 5 Hz low-pass filter. It is worth noting that to not alter the values of the windows when filtering, we saved the original values before performing the analysis. Finally, the algorithm identified filtered windows with an amplitude higher than 0.5 mV in order to find electrode pops that were not previously removed (see Fig. 7). In case at most 10% of channels (two channels) contained electrode pops, the original window was interpolated. Otherwise, it was maintained. It is worth noting that this method was not considered at the beginning of our preprocessing methodology. However, after analysing some ICs, we noticed that some segments contained one IC only for the electrode pops. Therefore, we added this step to reduce those outputs.

#### Interpolation of noisy EEG channels, preparation for ICA, and ICA processing

Finally, the channels selected to be interpolated in the phase *Removal of Noisy EEG Segments* were interpolated using the spherical interpolation method^33^, available in EEGLAB toolbox^34^. Finally, all segments were re-referenced to the average reference and decomposed by extended infomax ICA algorithm^35^.

From the original 648 hours of signal, 35.32 hours were removed by the algorithm due to experimental errors. Therefore, the dataset is based on 612.68 hours of EEG data.

### Independent components classification

**Fig. 8:**
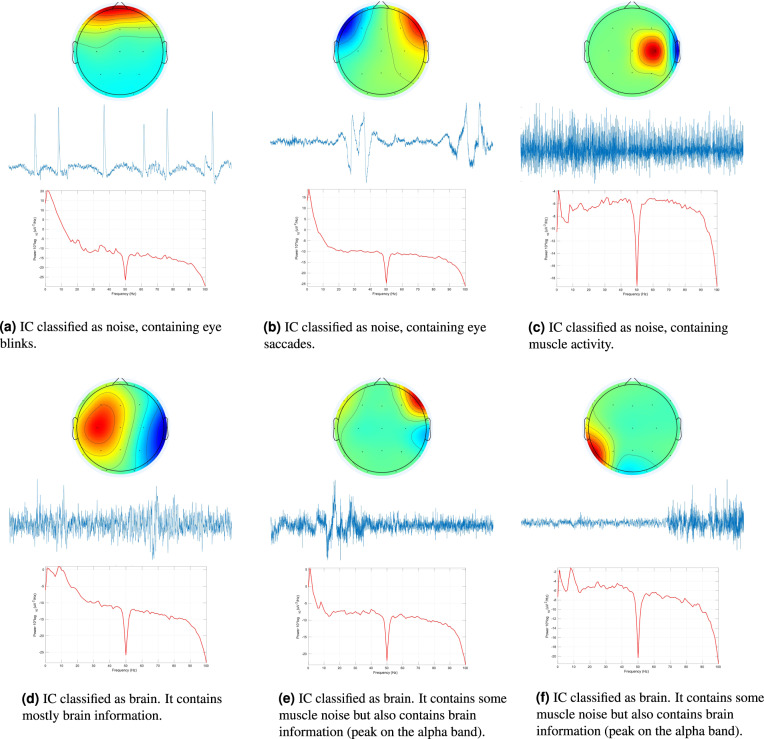
Time-series, topographic maps and power spectrum densities of example independent components. The presented time-series comprises only 5 seconds of the entire IC time-series.

The 612.68 hours of EEG data comprise 77,426 ICs. These data were randomly split into training and test sets. The training set contains 61,092 (78.86%) ICs, from 20 patients, whereas the test set contains 16,334 (21.14%) ICs, from the remaining 5 patients. Two experts visually inspected these segments. This analysis was performed following a semi-supervised approach using the ICLabel toolbox^12^ available in the EEGLAB^34^. The ICs were first automatically classified by the ICLabel classifier, as brain component or artifact, and then corrected by the experts, when needed. To make the corrections, they verified the IC time-series, power spectrum density (PSD), and topographic map of each IC. If they did not agree with the ICLabel classification, they would change it according to their analysis. Figure 8 presents some examples of analysed ICs. ICs presented in Fig. 8a–c predominantly present artifacts. Figure 8d shows an IC with brain information manifesting (i) on the dipole showing in the topoplot and (ii) on the alpha-band peak showing in the PSD spectrum. Figure 8e,f present ICs containing both brain and noisy data. Despite the existence of noise, experts classified both components as brain in order to maintain neural information that could still be useful in further analysis.

Training and test sets were differently analysed. Each IC of the training set was only inspected by one expert, i.e. if one expert already analysed one IC it was not examined by the other expert. This analysis method was performed to have a dataset classified following different opinions, especially on the doubtful ICs. Training set contains 43,038 (70.44%) brain ICs and 18,054 (29.56%) artifact ICs. Concerning the test set, it was firstly reviewed by both experts independently and, finally, the ICs with different classifications were discussed by them to assign a final classification. This approach was made to have a test set validated by both experts with the minimum possible subjectivity. Test set contains 11,437 (70.02%) brain ICs and 4,897 (29.98%) artifact ICs.

After all the processing methods, we extracted the time-series, PSDs and topographic maps of all ICs. IC time-series were obtained from the multiplication of the ICA weights with the EEG data. Depending on how long they are, these may contain from 2,560 to 153,600 samples. The IC PSDs were obtained using the *spectopo* function available in the EEGLAB toolbox. These were restricted to frequencies between 1 and 90 Hz in order to reject the effects of the used 0.5–100 Hz band-pass filter. The IC topographic maps were obtained using the *topoplot* function available in the EEGLAB toolbox. These comprise 67 × 67 pixels. Both PSD and topographic maps were normalised using the maximum and minimum values. The IC time-series were not normalised because experts reported that the amplitude of the data was used to decide if the IC should be removed in doubtful cases.

## Data Records

**Fig. 9:**
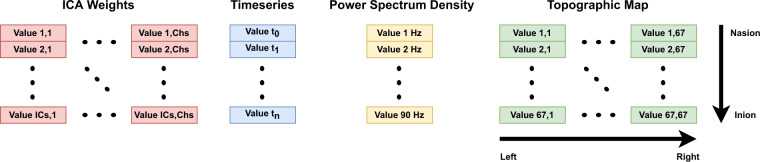
Example of text files containing ICA weights, IC time-series, PSD, and topographic map. The values of the ICA weights are stored using the number of ICs as rows and the number of EEG channels as columns. The values of the time-series are stored chronologically. The values of the PSD begin at 1 Hz and finish at 90 Hz. The values of the topographic map are stored as a square being the upper-left corner the left-nasion, the upper-right corner the right-nasion, the lower-left corner the left-inion and the lower-right corner the right-inion.

The dataset comprises two main directories containing the training and test sets. Inside each directory, three subdirectories contain artifact samples, not-artifact samples, and independent component analysis (ICA) weights. Data is stored in text files. Artifact and not-artifact subdirectories include the IC time series, the power spectrum density (PSD), and the topographic map. The files comprising the IC time-series and the PSD contain one-dimensional arrays, whereas those comprising topographic maps contain two-dimensional arrays. ICA weights subdirectory contains the weights used to convert the electroencephalogram (EEG) segments into ICs. These comprise two-dimensional matrices with the number of rows equal to the number of ICs and the number of columns equal to the number of EEG channels. Figure 9 presents how the data is stored in the files. The dataset is available at 10.5281/zenodo.6620655^36^.

## Technical Validation

The data was verified by F.L. with the purpose of finding whether there were corrupted samples. This dataset was used in Lopes *et al*.^27^. Authors reported 92.84% of sensitivity and 93.82% of specificity for the proposed model. No errors were found in the data during the study. This paper concluded that these data could be used for training new independent component classifiers. However, the authors encourage future users to report errors that they may find while producing their studies. These reports should be sent to the corresponding author in order to update the dataset.

## Data Availability

The custom code used to create this dataset is available on 10.5281/zenodo.6620655^36^.
